# With Great Hopes Come Great Expectations: Access and Adoption Issues Associated With COVID-19 Vaccines

**DOI:** 10.2196/26111

**Published:** 2021-08-04

**Authors:** Zhaohui Su, Dean McDonnell, Ali Cheshmehzangi, Xiaoshan Li, Daniel Maestro, Sabina Šegalo, Junaid Ahmad, Xiaoning Hao

**Affiliations:** 1 Center on Smart and Connected Health Technologies, Mays Cancer Center, School of Nursing UT Health San Antonio San Antonio, TX United States; 2 Department of Humanities Institute of Technology Carlow Carlow Ireland; 3 Department of Architecture and Built Environment University of Nottingham Ningbo China Ningbo China; 4 Hiroshima University Hiroshima Japan; 5 Program of Public Relations and Advertising Beijing Normal University-Hong Kong Baptist University United International College Zhuhai China; 6 Department of Environmental Health Institute for Public Health of Federation Bosnia and Herzegovina Sarajevo Bosnia and Herzegovina; 7 Department of Microbiology, Faculty of Medicine University of Sarajevo Sarajevo Bosnia and Herzegovina; 8 Prime Institute of Public Health Peshawar Medical College Peshawar Pakistan; 9 Division of Health Security Research, China National Health Development Research Center, National Health Commission, P.R. China Beijing China

**Keywords:** COVID-19, coronavirus, COVID-19 vaccine, made in China, vaccine efficacy, vaccine safety, vaccine, China, expectation, safety, efficacy, infectious disease, public health, consequence, public health, standard

## Abstract

Although COVID-19 vaccines are becoming increasingly available, their ability to effectively control and contain the spread of the COVID-19 pandemic is highly contingent on an array of factors. This paper discusses how limitations to vaccine accessibility, issues associated with vaccine side effects, concerns regarding vaccine efficacy, along with the persistent prevalence of vaccine hesitancy among the public, including health care professionals, might impact the potential of COVID-19 vaccines to curb the pandemic. We draw insights from the literature to identify practical solutions that could boost people’s adoption of COVID-19 vaccines and their accessibility. We conclude with a discussion on health experts’ and government officials’ moral and ethical responsibilities to the public, even in light of the urgency to adopt and endorse “the greatest amount of good for the greatest number” utilitarian philosophy in controlling and managing the spread of COVID-19.

## Background

How would we as a society remember COVID-19 after the postpandemic realities become the new normal? The destruction it caused, or the construction it motivated? The fears it inflamed, or the hopes it inspired? The transmission it kindled, or the determination it fanned? The sickness it roused, or the human solidarity it helped cement? As COVID-19 is still evolving, it might be difficult to determine how the pandemic might fold or the contours of the finale. What is clear, though, is that COVID-19 vaccines have already forged and will continue to shape society’s collective memories of the great pandemic of the 21st century—the almost-stopped-the-world-go-round global crisis that the COVID-19 pandemic is [1-3]. As of May 24, 2021, COVID-19 has already claimed 167 million infections and 3.46 million deaths [1]—numbers that might only represent a fraction of the true toll, as indicated by investigations led by the World Health Organization (WHO) and other organizations (eg, the Economist) [2,3]. Although the pandemic has upended the lives and livelihoods of thousands of millions of families and has dragged the world economy into unknown terrain [4], COVID-19 vaccines offer rays of hope that continue to draw people closer to the end of the tunnel [5].

Determined to build some “normalcy,” a global race to develop vaccines that can halt the pandemic has elevated on decades of experience and knowledge on immunization, the most advanced establishment of infrastructure, and an unwavering talent and motivation united to curb the spread of the virus [6,7]. Starting from December 2020, nine months after the WHO first labeled COVID-19 a global pandemic [8], the United Kingdom became the first nation to roll out mass vaccination [9]. Owing to its success in administering shots at the arms, as of May 2021, after months of strict social distancing mandates and within the confines of certain rules, UK residents have once again been able to enjoy shots at pubs indoors [10], with the company of strangers, friends, or one’s inner peace. COVID-19 vaccines, essentially, are the shots of hope people have been anxiously waiting for; when human contact is no longer as contagious as it used to be, hugs, handshakes, and heart-shaped selfies will become possible again across the world. Not to mention the happiness experienced when reuniting with families through nursing home visits, rekindling friendships with face-to-face lectures, and the bittersweet dash to a closing gate for business and leisure travel.

However, it is important to note that COVID-19 vaccines are not equally distributed silver bullets. How well COVID-19 vaccines can help curb the pandemic is contingent upon factors ranging from vaccine accessibility and vaccine efficacy to vaccine hesitancy, particularly in light of uncertainties associated with COVID-19 mutations [11-15]. Not much is discussed about critical issues associated with COVID-19 vaccine access and adoption while sharing positive news on COVID-19 vaccines and during talks about recovery and normalcy. Therefore, in this paper, we examine key factors that shape people’s access to and adoption of COVID-19 vaccines. Furthermore, we draw insights from the literature and aim to identify strategies that could boost people’s adoption of and the availability of COVID-19 vaccines, and ethical considerations associated with these strategies.

## Issues Associated With Vaccine Inequity and Accessibility

It is important to note that vaccine availability does not equate to vaccine accessibility [11]. As a result of limitations in vaccine production, although more COVID-19 vaccines will become available in the coming months, not all people will have the same level of access. In the United Kingdom, for instance, older adults and frontline workers (eg, health care professionals) will be vaccinated first [16]. Simultaneously, in the United States, vaccine distribution policy will be heavily influenced by federal and state policies [17]. In addition to the prioritized distribution of vaccines, trial data availability also affects COVID-19 vaccine accessibility to individual end-users. For example, although expectant mothers are susceptible to COVID-19 [18], most vaccines were not tested on pregnant or lactating women; these individuals will not have access to COVID-19 vaccines until data become available [19]. In other words, although COVID-19 vaccines are available to use, they are not available to use for everyone [19]. This revelation speaks volumes—even though women have been historically ignored and underrepresented in clinical trials [20], it is difficult to contemplate that the same gender inequality could occur amid a pandemic of COVID-19’s scale.

Equally disturbing, evidence further suggests that 90% of people living in 70 poor-income countries across the world will not have access to COVID-19 mass immunization campaigns until 2022 or 2023 [21,22], with the worst estimate pointing to 2024 [23]. On the other hand, high-income countries are hoarding vaccines; by early December 2020, Canada, for instance, had ordered enough doses of COVID-19 vaccines to inoculate each Canadian five times [24]. Overall, as of May 21, 2021, wealthy countries such as the United States, the United Kingdom, Australia, and other nations within the European Union (EU), have collectively ordered approximately 7.8 billion doses of COVID-19 vaccines, whereas only 270,200,000 vaccines are available for low-income countries [23].

COVID-19 vaccines often require advanced infrastructure for storage and delivery (see Table 1) [25]. For instance, to safeguard their potency, Pfizer-BioNTech vaccines are required to be stored and transported between –112°F and 76°F (–80°C to –60°C) [26]; this condition can only be achieved with advanced cold chain systems that are difficult to build and navigate [27,28]. In the United States, due to a failure in storage, a company responsible for vials of the Moderna vaccine, which must be kept cold, spoiled 890 doses destined for older adults in eight nursing home residents in Ohio [29]. At least in the United States, even though several states are not sharing their data, available evidence already shows that vaccine waste is prevalent across states [15]. Considering how higher-income countries face logistical issues using state-of-the-art and high-capacity cold chain systems [28], it is difficult to imagine how low- and middle-income countries will gain access to these vaccines, and how will they deliver these vials to their citizens. Furthermore, pressing issues such as accessibility of glass vials, syringes, and needles may further worsen the COVID-19 accessibility conundrum [28,30].

**Table 1:** Details of leading COVID-19 vaccines with known efficacy (as of June 2, 2021).

Name	Developer	Country	Type	Efficacy (Dose)	Status	Storage
Convidecia (or Ad5-nCoV)	CanSino	China	Adenovirus	65.28% (single dose)	Approved in China, emergency use in Chile, Hungary, Pakistan, etc	Stable in regular refrigerator for at least 6 months
BBIBP-CorV	Sinopharm	China	Inactivated	86% (2 doses, 3 weeks apart)	Approved in UAE^a^ and Bahrain; emergency use in Egypt and Jordan	Stable in regular refrigerator for at least 6 months
—^b^	Sinopharm-Wuhan	China	Inactivated	72.8%	Limited use in China and UAE	Stable in regular refrigerator for at least 6 months
CoronaVac (formerly PiCoVacc)	Sinovac	China	Inactivated	50.38%-78% (2 doses, 2 weeks apart)	Limited use in China, Brazil, etc	Stable in regular refrigerator for at least 6 months
Covaxin (or BBV152 A, B, C)	Bharat Biotech	India	Inactivated	78% (2 doses, 4 weeks apart)	Emergency use in India, Philippines, Zimbabwe, etc	At least a week at room temperature
Sputnik V	Gamaleya	Russia	Adenovirus	91.4% (2 doses, 3 weeks apart)	Early use in Russia	Freezer storage
EpiVacCorona	Vector Institute	Russia	Protein	— (2 doses, 3 weeks apart)	Limited use in Russia and Turkmenistan	Stable in refrigerator for up to 2 years
Vaxzevria (or AZD1222/Covishield)	Oxford-AstraZeneca	UK^c^ and Sweden	Adenovirus	60%-90% (2 doses, 4 weeks apart)	Stopped use in Denmark and Norway; emergency use in UK, Lebanon, Canada, etc	Stable in regular refrigerator for at least 6 months
Ad26.COV2.S	Johnson & Johnson	US^d^	Adenovirus	57%-72% (1 dose)	Stopped use in Denmark and Finland; emergency use in US, the European Union, etc	Up to 2 years at –4°F (–20°C) or up to 3 months at 36-46°F (2-8°C)
mRNA-1273	Moderna	US	mRNA	94.5% (2 doses, 4 weeks apart)	Approved in Canada; emergency use in US, UK, etc	Stable in refrigerator for up to 30 days
NVX-CoV2373	Novavax	US	Protein	49.4%-89.3% (2 doses, 3 weeks apart)	—	Stable in regular refrigerator for at least 6 months
Tozinameran or Comirnaty or BNT162b2	Pfizer-BioNTech	US and Germany	mRNA	95% (2 doses, 3 weeks apart)	Approved in Canada, Saudi Arabia, UAE, Bahrain, and Kuwait; emergency use in UK, US, etc	Freezer storage only at –94°F (–70°C)

^a^UAE: United Arab Emirates.

^b^Not available.

^c^US: United States.

^d^UK: United Kingdom.

## Issues Associated With COVID-19 Vaccine Safety and Vaccine Hesitancy

Assuming everything goes as planned, COVID-19 vaccine efficacy will still be contingent upon the abilities of individual health facilities to administer their doses. Emerging concerns point to the fact that these institutions often vary in terms of safety protocols, equipment maintenance, and staff training—critical competency criteria that could impact the vaccine administration process, and in turn, vaccine efficacy [17,31]. Competency of vaccine distribution centers also impacts end-user safety. For instance, in the state of West Virginia, 42 people who were scheduled to receive COVID-19 vaccines were mistakenly injected with an experimental monoclonal antibody treatment that should be administered via an intravenous infusion [32]. In reality, hospitals and medical centers across the world are overstretched and are at a breaking point in addressing the skyrocketing COVID-19 cases [33-36]; many further compound the moral (eg, who should receive COVID-19 vaccines?) and logistical (eg, how to administer these vaccines effectively and safely?) issues associated with vaccine administration.

After severe allergic reaction cases were first reported in the United Kingdom, regulators warned that Pfizer-BioNTech vaccine administration should not be carried out on people with a history of serious allergies [37]. It is worth noting that these reports occurred prior to the incidents of blood clots reported across the globe, especially in the EU nations [38]. The ever-emerging reports on COVID-19 vaccine side effects are alarming [39-41], as some individuals may not be aware of their allergies or underlying conditions that could expose them to severe vaccine side effects [11]. When they do, vaccine distribution facilities will have to face medical emergencies that they may or may not be capable of tackling. For the Pfizer-BioNTech vaccine trial alone, four volunteers developed Bell palsy or partial facial paralysis during the trial period [42]. For most established vaccines, such as seasonal influenza vaccines, allergic reactions often occur at a low rate estimated at one in a million people [43]; this number is substantially lower number compared to the current known allergic cases associated with COVID-19 vaccines, which is 11.1 per million people for the Pfizer-BioNTech COVID-19 vaccine [44].

In Norway, 23 older adults died shortly after COVID-19 vaccination [45]. Although the investigation is still underway, reports on vaccine side effects, especially if taken out of context, be it by legacy media outlets or conspiracy theory influencers, may further deepen the public’s fear, uncertainty, and distrust over COVID-19 vaccines [46]. Not to mention the tsunami of fact-based reports or how fake news may further exacerbate the public’s pandemic fatigue, along with potential mental health issues [47-49]. Inevitably, concerns associated with vaccine safety and reports on vaccine side effects may further hinder COVID-19 vaccine adoption [50-53], especially among those who spread unfounded vaccine rumors (eg, vaccine conspirators) or those who are already hesitant about COVID-19 vaccine uptake (eg, vaccine hesitants) [12]. Emerging reports on the impacts of COVID-19 mutations on vaccine efficacy may further compound the situation. Trial data on the Johnson & Johnson vaccines, for instance, show that although the vaccine efficacy is 72% in the United States, it dropped significantly in places where COVID-19 mutations are more prevalent—66% in Latin America and 57% in South Africa [54].

## Strategies to Promote COVID-19 Vaccine Accessibility and Adoption

### “Reimagining” COVID-19 Vaccine Doses to Improve Vaccine Accessibility

One way to increase vaccine accessibility that many governments are considering is by giving as many people as possible one dose instead of the original and approved two-dose vaccination regimen for fewer people [55]. Britain, for instance, along with other European countries [56], has already delayed administering the scheduled second doses of COVID-19 vaccines on the ground that “vaccinating a greater number of people with a single dose will prevent more deaths and hospitalizations than vaccinating a smaller number with two doses” [55]. In addition to delaying the administration of the second vaccine dose and capitalizing on vaccine overfill (ie, some Pfizer-BioNTech vaccine vials were found to contain a greater amount of the vaccine dose than expected), a surprise that many health care professionals are happy to unveil [57], epidemiologists are also weighing in the option of cutting COVID-19 vaccine doses in half (ie, from 100 mg to 50 mg), hoping to double the available Moderna vaccine supply in a timely fashion [58].

### “Extra” Doses or “Expected” Doses?

Although all the abovementioned measures could help health experts and government officials to capitalize on available vaccine doses, they each come with their own sets of caveats. Among all three measures, the least problematic approach is probably leveraging the vaccine overfill issue. However, even this approach has issues. The first problem lies in the knowledge and experience needed to extract extra doses from the vaccine vials. COVID-19 vaccines are fancy magic delicately packaged in tiny glass vials—they are exceedingly expensive in the way they are designed, developed, delivered, and deployed with care, or lack thereof [11]. The vaccine extraction procedures require medical expertise and special equipment to succeed, which could be an issue considering that hospitals in worst-hit places are often stretched thin. The particular syringe needed for the procedure is in short supply [59]. The second issue is rooted in Pfizer-BioNTech’s very business-minded calculations. Not wishing to break its Big Pharma stereotypes, Pfizer will deliver fewer numbers of vaccine vials to account for the difficult-to-extract “extra” doses—Pfizer’s contractual agreement with the US government counts doses, rather than vials [60].

In other words, health care professionals in the United States may soon have to extract the “expected” doses from each Pfizer-BioNTech vaccine vial. It is important to incentivize businesses, especially powerful Big Pharmas, amid COVID-19 to contribute to social goods. However, particularly in light of mechanisms such as the Defense Production Act of 1950 [61], it is questionable whether financial incentives are the only approaches governments can use. When all members of the public have to follow the COVID-19 safety measures, such as the United Kingdom’s waves of lockdowns, or get fined or jailed, for the greater good, then why are Big Pharma companies such as Pfizer-BioNTech not expected to do the same? Perhaps rather than arguing with governments about wording, dosing, and business bottom lines, Big Pharma companies like Pfizer-BioNTech should focus on producing more COVID-19 vaccines. Overall, it is not a sustainable approach to allow Big Pharma to see lucrative financial benefits in pandemics; societies at large have too many of these already for them to secure their astronomical bonus payments, ranging from the obesity epidemic, HIV epidemic, cancer epidemic, to communicable disease epidemics such as the annual seasonal influenza epidemics.

### Utilitarianism Without Consequentialism?

For the approaches that disregard the originally and only clinically tested and approved sets of dosing guidelines, both the problems and solutions may be substantially more challenging to obtain. Essentially, the splitting doses (ie, getting more people to receive one dose of COVID-19 vaccine) and halving doses (ie, getting more people to receive at least some dose of COVID-19 vaccine) methods are a manifestation of “the greatest amount of good for the greatest number” utilitarian philosophy developed by famed scholars such as John Stuart Mill [62]. These approaches have the potential to allow more people to have access to COVID-19 vaccines without actually improving COVID-19 production rates; however, an important caveat is that there is a lack of data on what might be the health consequences of administering one or halved dose of COVID-19 vaccines, rather than the clinically validated dosing regimen. Data on Pfizer-BioNTech vaccines already shows that the high threshold efficacy for single dose of COVID-19 vaccine is 52%. It could only reach the much-lauded 95% efficacy after the second dose is administered successfully within the prescribed time frame [63]. Available evidence from real-world mass vaccination in Israel further suggests that the actual efficacy of a single dose Pfizer-BioNTech vaccine may have a more disappointing number [64].

It is important to note that the statistics above only address the vaccine efficacy issue rather than other looming issues such as side effects and the interaction between coronavirus and vaccination. Some epidemiologists have already aired their concerns about the potential impacts of inoculating a large portion of the society with the same vaccine in a short time. Collectively, we have yet to figure out how coronavirus might evolve in light of these triggers; will a more potent and powerful variant of SARS-CoV-2 develop that is even more worrisome than the B.1.1.7 mutation first identified in the United Kingdom? It is important to note that some governments have already voiced their concerns over splitting and halving dosing COVID-19 vaccines. Even before data from Israel become available, making it the first country that has managed to vaccinate over 20% of its population and en route to inoculate the entire nation [65], the US Food and Drug Administration, for instance, warned public health officials of the danger associated with tempering with vaccine doses, citing that the idea is not supported by scientific evidence and “may ultimately be counterproductive to public health” [66].

A group of international advisers to the WHO, on the other hand, have recommended public health officials to follow the Pfizer-BioNTech vaccine schedule (ie, two doses given 3-4 weeks apart) rigorously when possible, but they have also suggested that countries with limited supplies of vaccines can consider delaying the second dose for up to 6 weeks [67]. It is important to note that initial evidence on dose splitting and extending intervals between shots is available from the AstraZeneca-Oxford trial [68]. Researchers found comparable efficacies between the two different time frames but disparate efficacies between dosages. Although these insights cannot be directly applied to mRNA vaccines developed by Pfizer-BioNtech and Moderna, they provide preliminary data on the interaction between dosing the vaccine efficacy, which should be further validated or updated by the mentioned effort undertaken by Moderna. Moreover, the Strategic Advisory Group of Experts on Immunization (SAGE), the committee that is tasked to advise WHO when it comes to immunization research and development (eg, COVID-19 vaccine guidelines) [69,70], recommended WHO and all health officials to follow Pfizer-BioNTech dosing and timeframe scheme as the group was reporting the results of their discussion on the approval of the WHO’s emergency use listing of Pfizer-BioNTech vaccines [71]. In extrapolation, then, it can be argued that it is recommended for officials to follow evidence-based schedules of the corresponding vaccines.

## Moral and Ethical Obligations in Public Health Policy-Making

Overall, considering the tsunami of information—fact-based or not—on COVID-19 vaccines, data are urgently needed to shed light on the safety and practicality of changing previously agreed-upon vaccine dosing regimens. Promisingly, a group of scientists in the United States is currently collecting and analyzing data on Moderna vaccines to evaluate the possibility of halving COVID-19 vaccine doses to increase vaccine accessibility [72]. It is essential to digest the fact that devising a different dosing schedule is different from squeezing an additional dose from COVID-19 vaccine vials due to overfilling; the former changes the clinically tested and validated guidelines, whereas the latter simply capitalizes on the fact that some glass vials contain more amount of vaccine.

Although the exact impact of changing the COVID-19 vaccine dosing schedule on personal and public health amid the pandemic is still unclear, what is clear is that governments need to make sure they base their decisions on scientific evidence rather than hopeful assumptions [73]. Yet, baseless assumptions, let alone politics, influencing any decision about COVID-19 vaccines could potentially impact thousands of millions of people’s lives and livelihoods. What is also clear is that, for people who have already received their first dose of COVID-19 vaccines, denying their access to the second dose is a blatant violation of informed consent, the very foundation of medical ethics, a baseline that should not be violated even in a time like the COVID-19 pandemic, particularly in light of dark events ranging from the Nazi’s medical experiments [74], Unit 731 atrocities [75], and the Tuskegee scandal [76]. Obtaining informed consent from potential vaccine receivers has been a tricky task [11], and the violation of informed consent—a contractual trust between individuals and health organizations and governments—may further exacerbate vaccination hurdles for all other immunization efforts.

## Conclusions

In this paper, we identified vaccine accessibility and adoption issues that can be collaboratively addressed by both private and public health sectors. Overall, more research is needed to shed light on these tasks, especially factoring in the ever-evolving nature of COVID-19 (eg, mutations) and phenomena such as “pandemic fatigue.” Great hopes have been invested in COVID-19 vaccines. However, it is important to understand that, for COVID-19 vaccines to effectively protect people from the pandemic, issues such as vaccine accessibility, vaccine efficacy, and vaccine hesitancy need to be solved first. In the context of COVID-19, great hopes will almost always mean great expectations—health experts and government officials have a fiduciary and an unwavering duty to the public to make sure they promise what can be delivered and they deliver what is promised. Although even rays of hope can light up the tunnel, in an environment where distrust is rampant, hope could be easily lost and difficult to rebuild.

## Abbreviations

EU: European Union
SAGE: Strategic Advisory Group of Experts on Immunization
WHO: World Health Organization
